# Safe Sialidase Production by the Saprophyte *Oerskovia paurometabola*: Gene Sequence and Enzyme Purification

**DOI:** 10.3390/molecules27248922

**Published:** 2022-12-15

**Authors:** Rumyana Eneva, Stephan Engibarov, Yana Gocheva, Simona Mitova, Alexander Arsov, Kaloyan Petrov, Radoslav Abrashev, Irina Lazarkevich, Penka Petrova

**Affiliations:** 1 Institute of Microbiology, Bulgarian Academy of Sciences, 1113 Sofia, Bulgaria; 2 Institute of Chemical Engineering, Bulgarian Academy of Sciences, 1113 Sofia, Bulgaria

**Keywords:** *Oerskovia paurometabola*, sialidase, gene sequencing, enzyme purification

## Abstract

Sialidase preparations are applied in structural and functional studies on sialoglycans, in the production of sialylated therapeutic proteins and synthetic substrates for use in biochemical research, etc. They are obtained mainly from pathogenic microorganisms; therefore, the demand for apathogenic producers of sialidase is of exceptional importance for the safe production of this enzyme. Here, we report for the first time the presence of a sialidase gene and enzyme in the saprophytic actinomycete *Oerskovia paurometabola* strain O129. An electrophoretically pure, glycosylated enzyme with a molecular weight of 70 kDa was obtained after a two-step chromatographic procedure using DEAE cellulose and Q-sepharose. The biochemical characterization showed that the enzyme is extracellular, inductive, and able to cleave α(2→3,6,8) linked sialic acids with preference for α(2→3) bonds. The enzyme production was strongly induced by glycomacropeptide (GMP) from milk whey, as well as by sialic acid. Investigation of the deduced amino acid sequence revealed that the protein molecule has the typical six-bladed β-propeller structure and contains all features of bacterial sialidases, i.e., an YRIP motif, five Asp-boxes, and the conserved amino acids in the active site. The presence of an unusual signal peptide of 40 amino acids was predicted. The sialidase-producing *O. paurometabola* O129 showed high and constant enzyme production. Together with its saprophytic nature, this makes it a reliable producer with high potential for industrial application.

## 1. Introduction

Sialidases (EC 3.2.1.18, acetylneuraminyl hydrolases, exo-alpha sialidases, neuraminidases) are a large group of enzymes that catalyze the cleavage of sialic acids from carbohydrate components in glycoproteins, glycolipids, oligosaccharides, synthetic substrates or polysialic compounds. Sialic acids are nine-carbon amino sugars that occupy the terminal positions in glycans of complex macromolecules in higher animals. This position determines their constant participation in key interactions and processes such as the binding of ligands, the masking of receptors, the recognition of “self” and “foreign” by the immune system, and many others [1]. The synthesis and degradation of sialic compounds are in equilibrium, which is ensured by the enzymes of sialic metabolism, including sialidases in eukaryotic cells [2,3]. Some of the microorganisms that exist in close contact with higher animals (parasites, commensals) can assimilate sialic acids as a carbon and nitrogen source, which provides them with an advantage in adaptation and in the competition with other microorganisms [4,5,6,7]. They achieve the cleavage of sialic acid from glycoconjugates with the help of their own sialidases. Obligatory in the metabolism of higher animals [8], sialidases are relatively rare in microorganisms. Sialidases occur unevenly among bacteria, and closely related species or even strains of the same species may differ in the ability to synthesize them [9]. However, according to the BRENDA enzyme database (http://www.brenda-enzymes.org/; accessed on 10 November 2022), more than 100 species of prokaryotes contain sialidase genes. The best-studied bacterial producers of sialidase are pathogenic and causative agents of severe human infectious diseases—*Vibrio cholerae* O1 (cholera), *Clostridium perfringens* (gas gangrene), *Pasteurella multocida* (zooanthroponosis in various forms), *Streptococcus pneumoniae* (pneumonia and meningitis), *Corynebacterium diphtheriae* (diphtheria), *Salmonella typhimurium* (salmonellosis), etc. [10,11,12]. Sialidases play an important role in pathogenesis by providing nutrients and promoting bacterial colonization, adhesion, and biofilm formation. Bacterial sialidases are used as a tool for the structural analysis of glycocompounds in many biomedical experiments to affect surface cell structures, for the glycosylation of therapeutic proteins, and for the design of new drugs and therapies [13,14,15,16]. Therefore, proteomics-grade sialidases with various activities are highly demanded, and the biggest world commercial producers are Sigma-Aldrich (Merck KGaA, Darmstadt, Germany), Palleon^®^ Pharmaceuticals (Shanghai Henlius Biotech Inc., Shanghai, China), and Creative Enzymes^®^ (Shirley, NY, USA). In all these biotechnological productions, however, pathogenic sialidase overproducers, such as *C. perfringens*, *Str. pneumoniae*, *S. typhimurium*, and *V. cholerae*, are engaged [17,18]. The search for their non-pathogenic substitutes is of paramount importance for safety reasons and would facilitate the process of isolation and purification of these commercially important enzymes.

A very promising alternative is the isolation and selection of saprophytic strains belonging to the order *Actinomycetales*, which are also sialidase producers. Although conditionally pathogenic actinomycetes such as *Trueperella pyogenes* and *Gardnerella vaginalis* are also known [19,20], the ‘saprophytic’ sialidases are generally safe for humans and animals. Moreover, according to Tan et al. [21], *Oerskovia* ssp. are spread as indigenous actinomycetes in goats and form a beneficial association with the goat’s gut, similar to bifidobacteria from the same class *Actinomycetia*. Recently, we isolated the new strain *O. paurometabola* O129, an aerobe, mesophilic actinobacterium that produces a high amount of extracellular sialidase. The partial purification of the enzyme allowed the determination of temperature and pH optima, as well as the evaluation of the impact of metal ions on its activity [22]. Here, we report the complete purification of the first *O. paurometabola* sialidase, the identification and sequencing of the responsible gene, a full biochemical characterization of the electrophoretically pure enzyme, and a bioinformatic analysis to reveal more about this novel protein molecule.

## 2. Results

### 2.1. Purification of the Novel Sialidase Enzyme of O. paurometabola O129

The newly discovered sialidase producer *O. paurometabola* O129 showed high and relatively constant enzyme production in common Nutrient Broth, under aerobic conditions. The maximum production (31 U/mL) was observed around the 24th hour. The crude extracellular enzyme was subjected to stepwise purification, and the results of each purification step are presented in Table 1.

Sialidase purification included: (1) protein precipitation with (NH_4_)_2_SO_4_ to 100% saturation, dialysis, and concentration of the precipitate; (2) two steps of anion-exchange chromatography—DEAE cellulose column; (3) separation through a Q-Sepharose column using an FPLC system. Each purification step was followed by a further increase of the concentration on an Amicon device.

**Figure 1:**
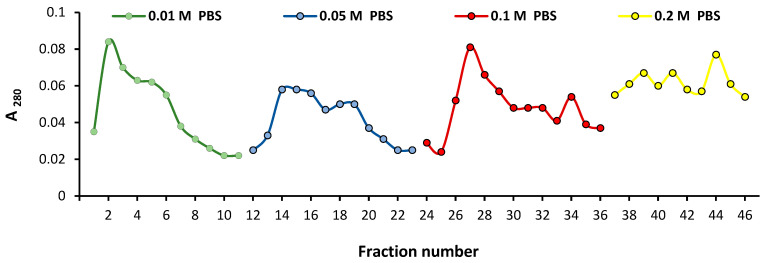
Elution profile of the anion-exchange chromatography of the sialidase from O129 on a DEAE cellulose column. The active peak was eluted with 0.1 M PBS at pH 7.5.

**Figure 2:**
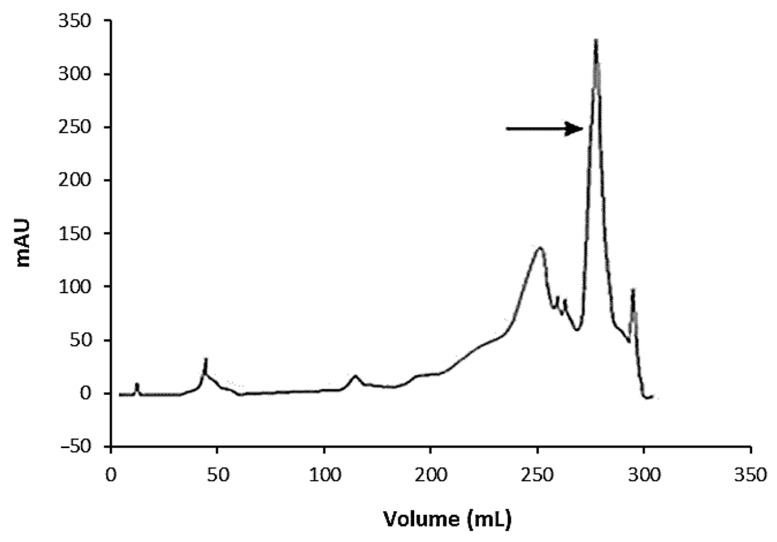
Elution profile of the sialidase from *O. paurometabola* O129 on a Q-Sepharose column in an FPLC system. The active peak is indicated by the arrow.

In the first step, a substantial part of the non-specific protein was removed, and an increase of the specific activity to 6800 U/mg of protein (22-fold purification) was achieved. With the DEAE cellulose column, the enzyme was eluted as a single peak with 0.1 M PBS, pH 7.5 (Figure 1). After its concentration, a preparation with a specific activity of 8200 U/mg was obtained. Further purification by ion-exchange chromatography on a Q-Sepharose column in an FPLC system followed by concentration led to an electrophoretically pure enzyme with a specific activity of more than 11,000 U/mg of protein. At this last stage, the enzyme was eluted to the greatest extent with a 70% salt solution in the elution buffer (Figure 2).

**Figure 3:**
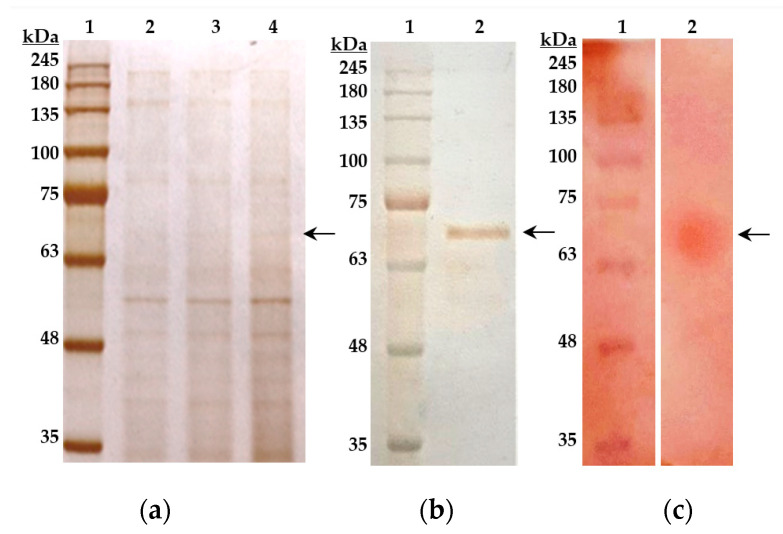
Sialidase of *O. paurometabola* O129 (marked by arrow), shown by SDS-PAGE in a 10% separating polyacrylamide gel. The enzyme was stained either by silver staining (**a**,**b**) or by the use of the Pierce™ Glycoprotein Staining Kit (Thermo Scientific Inc., Waltham, MA, USA), (**c**). Lanes and samples: (**a**): 1, molecular weight marker; 2–4, crude enzyme obtained after (NH_4_)_2_SO_4_ precipitation, dialysis, and purification through a DEAE cellulose column; (**b**): 1, molecular weight marker; 2, entirely purified sialidase of *O. paurometabola* O129; (**c**): 1, molecular weight marker; 2, purified sialidase after glycoprotein staining. The characteristic magenta color, which the purified protein acquired as a result of Schiff base staining, reveals the very likely presence of a glycosyl moiety. As a molecular weight marker, we used the Perfect^TM^ Tricolor Protein Ladder (EURx, Gdansk, Poland). The determination of the molecular weight of the purified sialidase was performed by the estimation of the relative mobility factor (Rf). The decimal log value corresponding to this Rf is approximately 70 kDa.

The purity and homogeneity of the *O. paurometabola* O129 extracellular sialidase were demonstrated by SDS polyacrylamide gel electrophoresis using a 10% separating gel and silver staining (Figure 3a,b). In the purified sample, a single protein band was visualized, with an apparent molecular mass of about 70 kDa when compared to authentic molecular weight standards (Figure 3b).

### 2.2. Biochemical Characterization of the Enzyme

#### 2.2.1. Substrate Specificity

**Figure 4:**
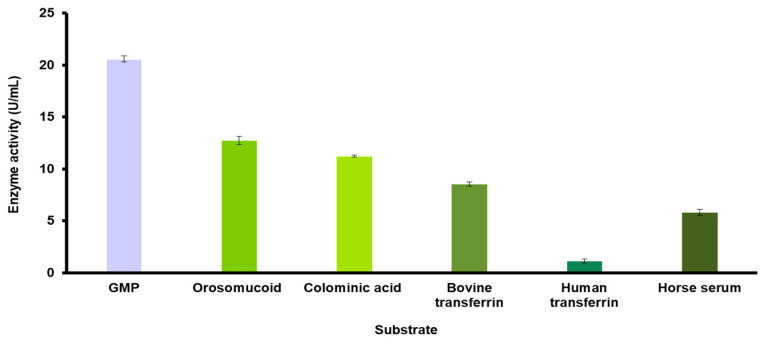
Substrate specificity of the sialidase isolated from *O. paurometabola* O129.

The purified sialidase from *O. paurometabola* O129 released sialic acid from diverse substrates (Table 2, Figure 4). Our results showed that it prefers to hydrolyze α(2→3) and α(2→8) bonds rather than α(2→6) linkages. However, the enzyme exhibited its highest activity towards the substrate glycomacropeptide (GMP), containing α(2→3,6) linkages. In addition, the Michaelis–Menten constant Km for GMP (1.33 mM) and the maximum velocity Vmax (96.15 µM/min·mg) of the O129 sialidase were determined.

#### 2.2.2. Induction

**Figure 5:**
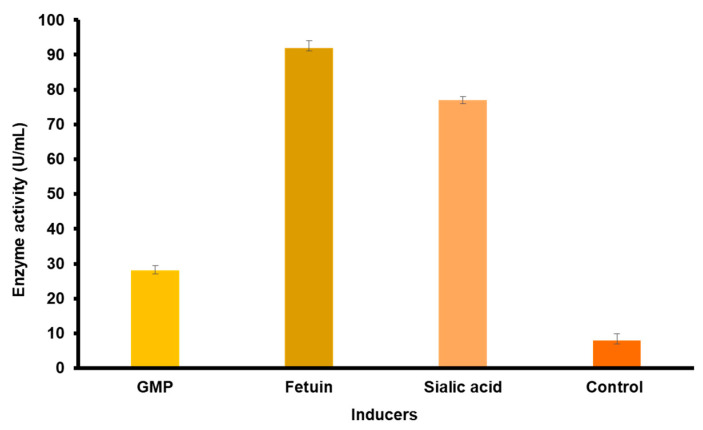
Effect of various inducers on the enzyme activity. The control sample was taken from a culture in a semisynthetic medium without inducer.

The observation of the effect of different inducers revealed that the highest enzyme activity could be achieved by the addition of fetuin (98 U/mL) and sialic acid (77 U/mL). The addition of GMP to the semisynthetic medium led to more than a 2-fold increase in enzyme activity (27.50 U/mL), as shown in Figure 5.

#### 2.2.3. Thermal Stability

**Figure 6:**
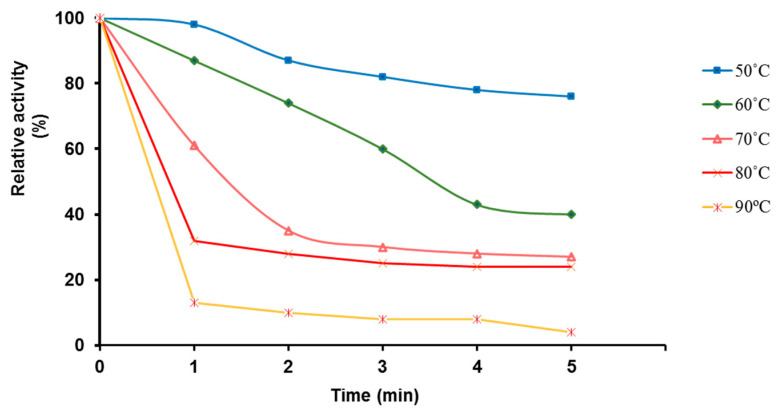
Thermostability of the sialidase from *O. paurometabola* O129. Each measurement point reflects the number of minutes during which the enzyme was subjected to the action of the corresponding temperature, after which it was cooled, and its activity was tested. Results are presented as percent residual activity relative to the activity of an unheated control enzyme sample.

Our results indicated that the O129 sialidase retained 80% of its activity after 5 min at 50 °C. After 3.5 min of heating at 60 °C, the activity was reduced by half, and approximately 30% of it was preserved at 70 °C and 80 °C. There still was some residual activity after the enzyme preparation was heated at 90 °C for 5 min (Figure 6).

#### 2.2.4. Stability at 37 °C

The use of sialidases in experiments on cell lines often requires prolonged contact of the enzyme with the cell surface, for example, for 72 h in a cytotoxicity test, at a temperature of 37 °C. Therefore, it is necessary to know whether the enzyme retains its activity at this temperature and for how long. A purified sample sterilized by a sterile filter was placed in a thermostat at 37 °C. Its activity was measured at the beginning of the experiment and after 24, 48, and 72 h. It was found that after 48 h, the activity of the purified enzyme was greatly reduced, indicating that the enzyme is not suitable for experiments that continue beyond this time.

### 2.3. O. paurometabola O129 Sialidase Gene Sequencing and Analysis

**Figure 7:**
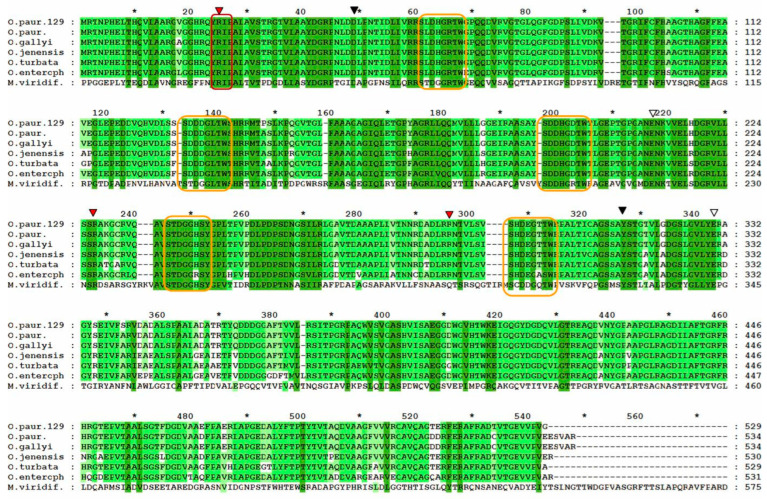
Amino acid sequence alignment of the novel *O. paurometabola* O129 sialidase (NCBI GenBank accession number OP429228) with the deduced amino acid sequences of the enzymes of *O. paurometabola* DSM 14281 (WP_204809048.1), *O. gallyi* strain Sa2CUA8 (WP_191790498.1), *O. jenensis* (WP_205305555.1), *O. turbata* (WP_030151436.1), *O. enterophila* (WP_068625153.1), and *M. viridifaciens* (WP_089006911.1). Full identity is outlined in dark green, partial identity, in green and light green, and lack of identity, in white. The Y/FRIP motif is enclosed in a red rectangle; the Asp-boxes are enclosed in yellow rectangles. The predicted conserved catalytic residues are marked with inverted triangles—red for the arginine triad R25, R227, R287, white for the glutamate E211 and E330, black for the aspartate D49 and tyrosine Y314.

**Figure 8:**
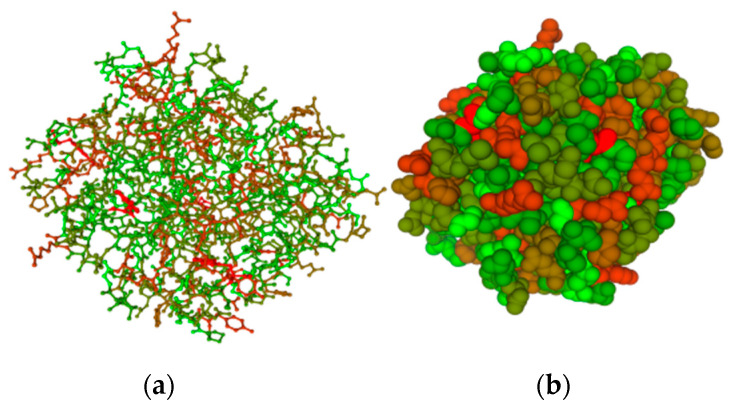
3D model of the sialidase of *O. paurometabola* O129 obtained by SWISS-MODEL Workspace [27]. (**a**) “Ball and stick” presentation of the chain; (**b**) “Space-full” model.

The gene encoding the sialidase enzyme of *O. paurometabola* O129 consists of 1589 bp and encodes a protein of 529 amino acids (aa). The open reading frame begins with the typical bacterial start codon ATG. The predicted molecular weight of the enzyme is 55,829 Da, with an isoelectric point pI 5.00. The amino acid homology search showed 98.67% similarity with a 534-aa putative glycoside hydrolase of *O. paurometabola* DSM 14281 (NCBI GenBank accession no. WP_204809048.1). To identify the precise conserved motifs and residues of the O129 sialidase, we compared its protein sequence to the respective sequences of five other *Oerskovia* species showing the highest identities (in descending order from 98 to 88% identity) and of the sialidase gene of the actinobacterium *Micromonospora viridifaciens*, whose amino acid sequence was thoroughly analyzed by Gaskell et al. [27]. The alignment is shown in Figure 7. *O. paurometabola* O129 sialidase gene possesses a YRIP motif Tyr-Arg-Ile-Pro, located near the N-terminus. The Asp-boxes are five: four with the Ser-X-Asp-X-Gly-X-Thr-Trp motif, and one with a Ser-X-Asp-X-Gly-X-Ser-Tyr motif. The putative arginine triad in O129 sialidase was defined as R25, R227, and R287. The conserved aspartate, glutamate, and tyrosine active site residues are supposed to be D49, E211, E330, and Y314 (Figure 7). The spatial model of the sialidase molecule provides insight into the arrangement of the 3D chain (Figure 8).

Using the SignalP-5.0 prediction program, a signal peptide of 40 amino acids was predicted in the sialidase of *O. paurometabola* O129, which indicates an unusual secretion mechanism.

## 3. Discussion

In the present study, the isolation of a sialidase from *O. paurometabola* in an electrophoretically pure preparation is presented for the first time. After culturing the producer strain O129 for 24 h in a common nutrient medium, the sialidase activity reached levels comparable to those of our previously described strain V13 of the typical sialidase producer *V. cholerae* [28]. We used a simple laboratory purification scheme to obtain a homogenous enzyme preparation with a high specific activity. Thus, we were able to determine the molecular weight of the mature enzyme. There is a great variety in the molecular weight of bacterial sialidases. The masses of their monomers are in the range of 40–150 kDa, and two groups are observed—small proteins with a molecular weight of about 42 kDa and large ones of about 60–70 kDa [16,29]. Bacterial sialidases are usually monomers, but there are enzymes consisting of two (*C. chauvoei*) or three subunits (*Clostridium septicum*, *Bacteroides fragilis*) [29,30]. Sometimes, a sialidase is part of a larger protein complex, as is the case in *Streptococcus oralis* [31].

The zymogram in native PAGE performed by us earlier [22] displayed only one active band for the enzyme from *O. paurometabola* O129. The SDS PAGE of the purified enzyme preparation in this study revealed also one distinct protein band, which corresponded well to the zymogram [22]. Apparently, the sialidase produced by the strain O129 consists of one monomer and belongs to the so-called large sialidases.

The molecular weight of the enzyme determined by electrophoresis showed a significant difference compared to that determined according to the predicted amino acid sequence—70 kDa and 56 kDa, respectively. We hypothesize that the higher Mw estimated by SDS-PAGE was due to the glycosylation of the protein, as can be seen in Figure 3c. This posttranslational modification was at first thought to be characteristic only of eukaryotes, where the glycosylation mechanism and functions are well studied. However, the number of recognized bacterial protein glycosylation systems continues to grow. Such have been found, for example, in *Helicobacter*, *Campylobacter*, and *Aeromonas*, as well as in Gram-positive *Clostridia* and *Listeria* for flagellin glycosylation, in *Escherichia coli* for adhesins modification, etc. [32]. Our previous studies on *Vibrio cholerae* non-O1/13 sialidase showed that this enzyme is a glycoprotein [33].

Bacterial sialidases can catalyze the hydrolysis of terminal sialic acids linked by the α(2→3), α(2→6), or α(2→8) linkages to a diverse range of substrates. In addition, some of these enzymes can catalyze the transfer of sialic acids from sialoglycans to asialoglycans via a transglycosylation mechanism [16]. The O129 enzyme showed lower activity towards substrates such as bovine and human transferrin containing only α(2→6) bonds. Similar results were reported for *C. diphtheriae* sialidase, which prefers the α–acid glycoprotein (orosomucoid, α(2→3), α(2→6) linkages) over transferrin (α(2→6) linkages) [24]. It should be noted that the O129 enzyme degraded colominic acid (homopolymer of α(2→8) bounded sialic acids) with relatively high efficiency. Most of the bacterial sialidases are weakly active or completely inactive towards this compound [28,34]. The lower activity towards horse serum could be explained by the presence of sialic acids that are O-acetylated, a modification known to reduce the degree of hydrolysis by sialidases [26,35]. Considering that sialidase is the enzyme that carries out the first step in the catabolism of sialoconjugates, which are found almost entirely in the animal world, we can assume that its saprophytic producers among actinomycetes contribute to the rapid degradation of these substances. On the other hand, sialic acid may be used as a source of carbon and nitrogen in these organisms; thus, its uptake gives them an advantage in their adaptation to the environment with respect to microorganisms that do not possess this property. In pathogenic sialidase producers, the enzyme is a factor that promotes invasion and colonization of the host, which, according to Corfield [10], is actually a consequence of its trophic function. An advantage of saprophytic producers of sialidase is that they can become a factor of pathogenicity only in very rare cases, in patients with a severely compromised immune system. Regarding *O. paurometabola*, as far as we know from the literature, no clinical cases of infections with this microbial species have been described, which makes O129 extremely suitable as an enzyme producer on an industrial scale. A technology for the degradation of azo dyes by a strain of this species was recently published, i.e., the organism was tested in purification technologies on a large scale, without posing any risk to humans [36].

Our results indicate the inducible nature of the *O. paurometabola* O129 sialidase synthesis. Sialoglycoconjugates such as fetuin, transferrin, etc., have been described as effective inducers of sialidase production in bacteria [10]. Sialic acid has a positive influence on enzyme production in *Pseudomonas aeruginosa*, *Str. pneumoniae*, *V. cholerae* non O1/13, *Aeromonas* sp. A40/02, *C. perfringens* [28,37,38,39,40]. The addition of GMP to the semisynthetic medium leads to more than a 2-fold increase in enzyme activity. The inductive effect of this compound was documented in the synthesis of sialidases from *Erysipelothrix rhusiopathiae*, *V. cholerae* non O1/13, and *Aeromonas* sp. A40/02 strains [28,39,41].

Sialidase synthesis in *O. paurometabola* O129 was stimulated by compounds containing both glycosidically linked sialic acids and free sialic acid, which is a product of the sialidase reaction. It is known that enzyme production is generally induced by the presence of the susceptible chemical bond (in our case, the α-glycosidic bond) as well as of one of the compounds joined by this bond (the sialic acid). The inductive secretion of the sialidase from *O. paurometabola* O129 presents an opportunity to achieve a significantly higher production when the nutrient medium is optimized by supplementing it with an inducer.

Usually, thermostability correlates with the temperature optimum. Our results showed a different trend in the effect of temperature on *O. paurometabola* O129 sialidase. Although the temperature optimum of the enzyme is 37 °C [22], heating it to 50–90 °C for 1 to 5 min resulted in a significant loss of activity, which was gradual but did not lead to its complete loss. In this respect, the O129 enzyme is more thermostable than the *C. perfringens* sialidases NanJ and NanH [42]. A loss of enzyme activity of approximately 80% occurred after incubation of these enzymes for 5 min at 60 °C. At the same time, the O129 enzyme appeared to be more thermosensitive than the sialidases from *Arcanobacterium pyogenes* and bifidobacteria [34,43]. The first of them preserved approximately 45% of its activity after 1 h at 75 °C, while the bifidobacteria enzyme demonstrated nearly full activity after incubation at 80 °C for 30 min.

**Figure 9:**
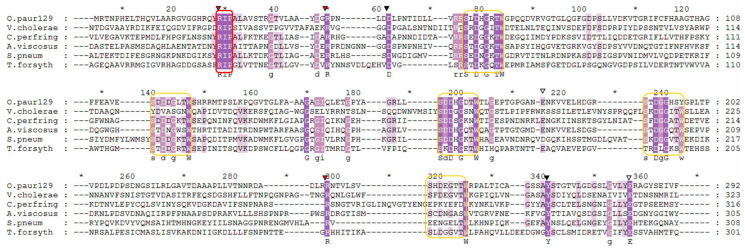
Amino acid sequence alignment of the novel *O. paurometabola* O129 sialidase (NCBI GenBank accession number OP429228) with the deduced amino acid sequences of the enzymes of *V. cholerae* (AWB74302.1), *C. perfringens* (APZ74275.1), *A. viscosus* (VEI16517.1), *Str. pneumoniae* (WP_129559156.1), and *Tannerella forsythia* (CQ24155.1). Full identity is outlined in dark violet, partial identity, in light violet, and lack of identity, in white. The Y/FRIP motif is enclosed in a red rectangle; the Asp-boxes are enclosed in yellow rectangles. The predicted conserved catalytic residues are marked with inverted triangles—red for the arginine triad, white for glutamate, and black for aspartate and tyrosine.

Sialidases of eukaryotic and prokaryotic origin have a similar mechanism of action and therefore display a high similarity in the conserved amino acid residues in their active center. Regardless of that, the similarity in their complete amino acid sequences is relatively low—only about 30% [16]. Non-viral sialidases share some common structural features such as the [Y/F] RIP motif, Aspartate boxes (Asp-boxes), and specific amino acids in the active site (Figure 9). The latter include three arginine residues (the arginine triad, which interacts with the carboxyl group of the Neu5Ac substrate [44] and several other specific residues that enhance the catalytic function (Figure 9). All of these conserved elements are present in the O129 sialidase (Figure 7). It carries a YRIP motif, which is considered to be involved in the enzymatic reaction, as its arginine residue is part of the arginine triad (R25, R227, R287) in the active site. Corresponding to the additional conserved residues of *M. viridifaciens* sialidase active center [27], and therefore suggesting to facilitate the catalytic process in O129 sialidase, are the glutamate (E211, E330), tyrosine (Y314), and aspartic acid (D49) residues. The Asp-boxes are five, and in bacterial sialidases, they are usually found in three to five copies, remote from the active site, in topologically equivalent locations. So far, there is no proven specific role of the Asp-boxes, but there are several assumptions that might be relevant to the present case. Eight protein families were found to contain sequences significantly similar to known Asp-boxes, which are among the most highly conserved sequence motifs, thus suggesting their functional importance [45]. Authors studying sialidase proteins’ structures suppose these elements may keep the proper folding of the protein chain, as, for example, the five Asp-boxes of *M. viridifaciens*, *V. cholerae,* and *S. typhimurium* occur in topologically equivalent positions at the turn between the third and the fourth strands of each sheet in the propeller arm [27,46]. An alternative explanation of their putative structural role in different protein families was mentioned by Copley et al. [45], who proposed the formation of energetically favorable conformations. The same authors suggested it may be significant that Asp-box motifs reside mostly in secreted proteins, except for cytosolic sialidases and sulfite oxidases. It is also worth noting that Asp-boxes occur frequently in proteins that act on, or interact with, polysaccharides. Polysaccharides are often substrates of Asp-box-containing glycosyl hydrolases such as sialidases. It is thus possible that Asp boxes bind polysaccharides [45].

**Figure 10:**
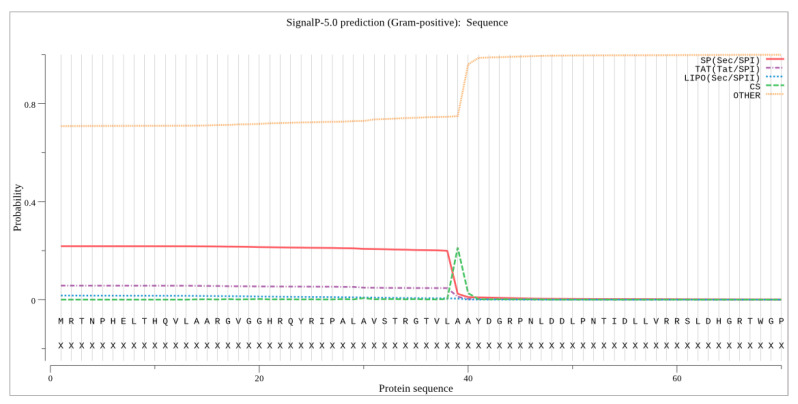
Signal peptide of *O. paurometabola* O129 sialidase as predicted with high probability by SignalP 5.0 software. The cleavage site LAA is marked by a green peak.

Usually, bacterial sialidases consist of a sialidase domain for catalytic activity and extra domains that facilitate substrate binding or enzyme localization [16]. However, the O129 sialidase is supposed to lack extra domains. Most bacterial sialidases are secretory proteins and contain signal peptides that are cleaved during the protein secretion process [16]. Some of them are membrane-bound enzymes possessing membrane-anchored domains [47]. Unlike all of them, the sialidase from *O. paurometabola* O129 contains a signal peptide whose sequence suggests “other” secretion mechanisms, different from the so-called ‘Sec’ and ‘Tat’ mechanisms (Figure 10).

The conserved sequence YRIP, which contains one of the residues of the arginine triad, falls within the signal peptide. This probably means that this peptide is not excised during the secretory process.

## 4. Materials and Methods

### 4.1. Bacterial Strain and Maintenance

*O. paurometabola* strain O129, obtained from a meat wash, was received from the collection of the National Center of Infectious and Parasitic Diseases (NCIPD) in Sofia, Bulgaria, and was identified by sequencing the 16S ribosomal gene. The strain was deposited in the National Bank for Industrial Microorganisms and Cell Cultures (NBIMCC) under Registration No NBIMCC 9093. The pure bacterial culture was maintained on Brain Heart Infusion broth (BHI), Nutrient Broth (NB), or Tryptic soy agar (TSA) at 30 °C and stored at −20 °C or −80 °C in 30% glycerol. For the purpose of sialidase isolation and purification, the producer *O. paurometabola* strain O129 was grown in NB, in 100 mL Erlenmeyer flasks (20 mL working volume) aerobically at 30 °C with agitation (160 rpm).

### 4.2. Isolation of DNA, PCR, and Sialidase Gene Sequence Analysis

The total DNA of *O. paurometabola* O129 was extracted from an overnight culture using a GeneJET Genomic DNA Purification Kit (Thermo Scientific™, Waltham, MA, USA). The PCR amplification of the gene was performed in a QB-96 Satellite Gradient Thermal Cycler (LKB Vertriebs GmbH, Vienna, Austria) with the primer pair OS_F1 (5′-ATG CGC ACG AAC CCC CAC GAA CTC ACC CAT CAG GT-3′) and OS_R (5′TCA GCC GAC CGG CAC GAC CTC CCC GGT CACGGT GT3′). The PCR mixture consisted of 50 ng of DNA template, 0.4 µM primers, Premix Ex Taq Hot Start Version (Clontech Laboratories, Inc., Takara Bio Company, Mountain View, CA, USA), and sterile water to a 25 µL final volume. The PCR reaction was performed under the following temperature profile: denaturation for 3 min and 30 sec at 98 °C; 38 cycles: 10 s denaturation at 98 °C, 45 s annealing at 68 °C, and 2.5 min elongation at 72 °C; final elongation for 5 min at 72 °C.

The sequencing of the PCR fragment was performed by Macrogen Inc. (Amsterdam, The Netherlands) with the above-listed primers and an additional primer OS_F2 (5′CGC ACG CAG GTG CGC GCC GCC GT3′).

The obtained sequences were processed by Chromas LITE, version 2.1, and CAP3 programs, while the comparison with the NCBI GenBank database was conducted by BLASTN. Sequence alignment and identity visualization were performed by the GeneDoc 2 program [48]. The assembled sequence was deposited in NCBI GenBank with accession number OP429228. The molecular modeling of the sialidase was performed in SWISS-MODEL Workspace [49]. Signal peptide prediction was carried out with SignalP 5.0 software (https://bio.tools, accessed on 1 November 2022).

### 4.3. Sialidase Activity Assay

The sialidase activity was measured quantitatively throughout the purification process by using the colorimetric thiobarbituric acid method of Uchida et al. [50]. The substrate for the determination of the enzyme activity was GMP, isolated by us from milk whey in laboratory conditions [51]. The protein part of GMP includes 59–61 amino acid residues, among which tyrosine, tryptophan, phenylalanine, and cysteine are missing. Its carbohydrate component contains galactose, galactosamine, and related sialic acids in a molar ratio of approximately 2:1:1. Variations in the glucoside and amino acid composition of GMP do not affect its use as a substrate for the determination of sialidase activity. The solution of 10 mg/mL of GMP contained 0.3 mg/mL of covalently linked sialic acids, equal to a 100 μM concentration of sialic acids [23,51]. One unit of sialidase activity is defined as the amount that releases 1 μmol of sialic acid (Neu5Ac) for 1 min under standard conditions using GMP as a substrate. The obtained protein was estimated by the Bradford method [52], using bovine serum albumin (BSA) as a standard.

### 4.4. Purification of the Extracellular Sialidase from O. paurometabola O129

The culture liquid was harvested, and the cells were removed from the culture of O129 by centrifugation at 1400 g for 20 min. The supernatant was gradually brought to 100% ammonium sulfate saturation and stored for 18 h at 4 °C. The precipitated proteins were collected by centrifugation at 1400 g for 30 min. The protein pellet was dissolved in a small volume of saline solution and dialyzed against distilled water for 24 h at 4 °C. The dialyzed preparation was concentrated on an Amicon ultrafiltration cell with a Diaflo PM 30 membrane (Amicon Corporation, Lexington, MA, USA). It was loaded onto an anion-exchange chromatography column containing DEAE cellulose (Whatman Inc., London, UK). The column was washed successively with 0.01 M, 0.02 M, 0.1 M, and 0.2 M PBS, pH 7.5. Fractions of 2 mL were collected at a flow rate of 20 mL/h. The protein concentrations of column effluent fractions were estimated by measuring A_280_ on a UV/vis Specord spectrophotometer. The fractions providing protein peaks were united, concentrated in the Amicon ultrafiltration cell, and assayed for sialidase activity. The samples obtained by the first ion-exchange chromatography step were applied to a Hiprep QFF16/10 column containing a Q-Sepharose cationic resin in a General Electric Äktapurifier FPLC system. Elution was conducted by potassium phosphate buffer, pH 8.0, with an initial concentration of 0.01 M and the following concentration gradient from 0.4 to 0.6 M, supplemented with 2 M NaCl. Fractions of 5 mL were collected at a flow rate of 1 mL/min. Fractions providing protein peaks were united, concentrated, and assayed for enzyme activity. The active peak effluent was washed with pure buffer to remove salts.

### 4.5. SDS-PAGE and Glycoprotein Staining

Protein purity was monitored by SDS-PAGE. SDS-PAGE was performed with a 10% (*w*/*v*) separating gel and a 4% (*w*/*v*) concentrating gel. The purified sialidase was resolved at 100 V for 2.5 h [53]. Protein bands were visualized by silver staining performed according to the method described by Chevallet et al. [54]. The Perfect^TM^ Tricolor Protein Ladder (EURx, Gdansk, Poland) was used as a protein molecular weight standard. The determination of the molecular weight of the enzyme was performed by the relative mobility factor (Rf). In order to prove the glycoprotein nature of the O129 sialidase, we performed SDS-PAGE and stained the gel using the Pierce™ Glycoprotein Staining Kit for Protein Electrophoresis and Western Blotting (Thermo Scientific Inc., Waltham, MA, USA).

### 4.6. Enzyme Characterization and Kinetic Parameters

The substrate specificity of the extracellular sialidase of *O. paurometabola* O129 was examined with the following compounds, suspended in PBS, pH 5.5: human and bovine transferrin, orosomucoid (all three from Sigma-Aldrich Chemie GmbH, Steinheim, Germany), horse serum (Sigma-Aldrich), colominic acid (Koch-Light, Colnbrook Berks, England) and GMP. The kinetic parameters Km and Vmax were determined by the method of Lineweaver and Burk [55] for GMP as a substrate. Vmax was expressed as micromoles of sialic acid released in 1 min per milligram of enzyme.

### 4.7. Induction

The inductivity of neuraminidase production was studied using a semisynthetic medium, containing (in %) (NH_4_)_2_HPO_4_, 0.2; KH_2_PO_4_, 0.35; MgSO_4_, 0.015; NaCl, 0.5; yeast extract, 0.01; pH 8.0 Neu5Ac (Sigma-Aldrich Chemie GmbH, Steinheim, Germany); fetuin (Serva, Heidelberg, Germany) and GMP were tested as inducers in concentrations of 0.5%. Erlenmeyer flasks of 100 mL with 20 mL of semi-synthetic medium supplemented with the inducer and control flasks without the inducer were inoculated with an 18 h culture and cultivated on a rotary shaker at 30 °C. Two replicates were run for each inducer.

### 4.8. Thermal Stability

The thermal stability was determined by pre-incubating samples of the partially purified enzyme at temperatures of 50–90 °C, at 10 °C intervals and for different time intervals (0–5 min, every 1 min). The residual enzyme activity was determined under standard conditions for enzyme analysis, at 37 °C, which was the temperature determined during the experiments as the optimum for the enzyme’s action. A non-preheated sample was used as the control. The thermal stability at 37 °C was examined by placing a purified filter-sterilized sample in a thermostat. Sialidase activity was measured at the beginning of the experiment and after 24, 48, and 72 h.

## 5. Conclusions

The genetic and biochemical characterization of a novel sialidase was performed. The producer strain is a non-pathogenic saprophyte able to tolerate a wide range of media compositions, inducers, and cultivation conditions. The sialidase produced by *O. paurometabola* O129 is extracellular and with an activity comparable to that of sialidases from pathogenic producers. The present study is the first to confirm and purify a sialidase from *O. paurometabola* and thus sheds fresh light on the mechanisms of interaction of *Actinomycetes* with sialo-containing substrates. The new enzyme is reliable under various conditions and seems particularly promising for industrial production by a new and safe bacterial strain.

## Figures and Tables

**Table 1:** Summary of the purification of the extracellular sialidase from *O. paurometabola* O129.

Fraction	Volume (mL)	Enzyme Activity (U/mL)	Total Activity (U)	Protein (mg/mL)	Specific Activity (U/mg)	Purification (Fold)	Yield (%)
Culture liquid	50	31	1550	0,1	310	1	100
(NH_4_)_2_SO_4_ precipitation	5	136	680	0.02	6800	22	44
DEAE cellulose	1.3	328	427	0.04	8200	26	28
Q-Sepharose	1	227	227	0.02	11350	37	15

**Table 2:** Linkages that are attacked by sialidase enzymes in different substrates.

Substrate	Linkage	Reference
Glycomacropeptide (GMP)	α(2→3); α(2→6)	[23]
Orosomucoid	α(2→3); α(2→6)	[24]
Colominic acid (polysialic acid)	α(2→8)	[25]
Human transferrin	α(2→6)	[24]
Bovine transferrin	α(2→6)	[24]
Horse serum	α(2→3); α(2→6); O-acetylated sialic acids	[26]

## Data Availability

Not applicable.

## Sample Availability

Samples of the compounds are not available from the authors.

## References

[1] Schauer R., Kamerling J.P. (2018). Exploration of the Sialic Acid World. Adv. Carbohydr. Chem. Biochem..

[2] Giacopuzzi E., Bresciani R., Schauer R., Monti E., Borsani G. (2012). New insights on the sialidase protein family revealed by a phylogenetic analysis in metazoa. PLoS ONE.

[3] Eneva R., Engibarov S., Abrashev R., Krumova E., Angelova M. (2021). Sialic acids, sialoconjugates and enzymes of their metabolism in fungi. Biotechnol. Biotechnol. Equip..

[4] Roggentin P., Schauer R., Hoyer L., Vimr E. (1993). The sialidase superfamily and its spread by horizontal gene transfer. Mol. Microbiol..

[5] Vimr E.R. (2013). Unified theory of bacterial sialometabolism: How and why bacteria metabolize host sialic acids. ISRN Microbiol..

[6] Almagro-Moreno S., Boyd E.F. (2009). Insights into the evolution of sialic acid catabolism among bacteria. BMC Evol. Biol..

[7] Almagro-Moreno S., Boyd E.F. (2010). Bacterial catabolism of nonulosonic (sialic) acid and fitness in the gut. Gut Microbes.

[8] Minami A., Kurebayashi Y., Takahashi T., Otsubo T., Ikeda K., Suzuki T. (2021). The function of sialidase revealed by sialidase activity imaging probe. Int. J. Mol. Sci..

[9] Juge N., Tailford L., Owen C.D. (2016). Sialidases from gut bacteria: A mini-review. Biochem. Soc. Trans..

[10] Corfield T. (1992). Bacterial sialidases-roles in pathogenicity and nutrition. Glycobiology.

[11] Mizan S., Henk A., Stallings A., Maier M., Lee M.D. (2000). Cloning and characterization of sialidases with 2-6′ and 2-3′ sialyl lactose specificity from *Pasteurella multocida*. J. Bacteriol..

[12] Minami A., Ishibashi S., Ikeda K., Ishitsubo E., Hori T., Tokiwa H., Taguchi R., Ieno D., Otsubo T., Matsuda Y. (2013). Catalytic preference of *Salmonella typhimurium* LT2 sialidase for *N*-acetylneuraminic acid residues over *N*-glycolylneuraminic acid residues. FEBS Open Bio..

[13] Mariño K., Bones J., Kattla J.J., Rudd P.M. (2010). A systematic approach to protein glycosylation analysis: A path through the maze. Nat. Chem. Biol..

[14] Mountney A., Zahner M.R., Lorenzini I., Oudega M., Schramm L.P., Schnaar R.L. (2010). Sialidase enhances recovery from spinal cord contusion injury. Proc. Natl. Acad. Sci. USA.

[15] Solá R.J., Griebenow K. (2010). Glycosylation of therapeutic proteins: An effective strategy to optimize efficacy. BioDrugs.

[16] Kim S., Oh D.-B., Kang H.A., Kwon O. (2011). Features and applications of bacterial sialidases. Appl. Microbiol. Biotechnol..

[17] Merck products: Sialidase. https://www.sigmaaldrich.com/BG/en/search/sialidase?facet=facet_product_category%3Aenzymes&focus=products&page=1&perpage=30&region=global&sort=relevance&term=sialidase&type=product.

[18] Creative Enzymes® Products: Sialidase. https://www.creative-enzymes.com/search.html.

[19] Lee J.-Y., Seo S., Shin B., Hong S.H., Kwon E., Park S., Hur Y.M., Lee D.-K., Kim Y.J., Han S.B. (2022). Development of a new biomarker model for predicting preterm birth in cervicovaginal fluid. Metabolites.

[20] Rzewuska M., Kwiecień E., Chrobak-Chmiel D., Kizerwetter-Świda M., Stefańska I., Gieryńska M. (2019). Pathogenicity and Virulence of *Trueperella pyogenes*: A Review. Int. J. Mol. Sci..

[21] Tan H., Deng Z., Cao L. (2009). Isolation and characterization of actinomycetes from healthy goat faeces. Lett. Appl. Microbiol..

[22] Eneva R., Engibarov S., Gocheva Y., Mitova S., Petrova P. Novel sialidase from non-pathogenic bacterium *Oerskovia paurometabola* strain O129. *Z*. *Naturforsch*. *C*
**2022**. https://www.degruyter.com/document/doi/10.1515/znc-2022-0051/html.

[23] Neelima S., Sharma R., Rajput Y.S., Mann B. (2013). Chemical and functional properties of glycomacropeptide (GMP) and its role in the detection of cheese whey adulteration in milk: A review. Dairy Sci. Technol..

[24] Kim S., Oh D.-B., Kwon O., Kang H.A. (2010). Identification and functional characterization of the NanH extracellular sialidase from *Corynebacterium diphtheriae*. J. Biochem..

[25] Yamamoto T., Ugai H., Nakayama-Imaohji H., Tada A., Elahi M., Houchi H., Kuwahara T. (2018). Characterization of a recombinant *Bacteroides fragilis* sialidase expressed in *Escherichia coli*. Anaerobe.

[26] Wasik B.R., Barnard K.N., Ossiboff R.J., Khedri Z., Feng K.H., Yu H., Chen X., Perez D.R., Varki A., Parrish C.R. (2017). Distribution of O-acetylated sialic acids among target host tissues for influenza virus. mSphere.

[27] Gaskell A., Crennell S., Taylor G. (1995). The three domains of a bacterial sialidase: A beta-propeller, an immunoglobulin module and a galactose-binding jelly-roll. Structure.

[28] Eneva R., Engibarov S., Petrova P., Abrashev R., Strateva T., Kolyovska V., Abrashev I. (2015). High production of neuraminidase by a *Vibrio cholerae* non-O1 strain—The first possible alternative to toxigenic producers. Appl. Biochem. Biotechnol..

[29] Traving C., Schauer R. (1998). Structure, function and metabolism of sialic acids. Cell. Mol. Life Sci..

[30] Tanaka H., Ito F., Iwasaki T. (1992). Purification and characterization of a sialidase from *Bacteroides fragilis* SBT3182. Biochem. Biophys. Res. Commun..

[31] Byers H.L., Tarelli E., Homer K.A., Beighton D. (2000). Isolation and characterisation of sialidase from a strain of *Streptococcus oralis*. J. Med. Microbiol..

[32] Eichler J., Koomey M. (2017). Sweet New Roles for Protein Glycosylation in Prokaryotes. Trends Microbiol..

[33] Eneva R., Engibarov S., Sirakov I., Kolyovska V., Pavlova M., Petrov P., Nenova R., Abrashev I. (2017). Sialidase nanH of the non-toxigenic *Vibrio cholerae* strain V13 is a glycoprotein. Comptes Rendus L’académie Bulg. Sci..

[34] Kiyohara M., Tanigawa K., Chaiwangsri T., Katayama T., Ashida H., Yamamoto K. (2011). An exo-α-sialidase from bifidobacteria involved in the degradation of sialyloligosaccharides in human milk and intestinal glycoconjugates. Glycobiology.

[35] Frey A.M., Satur M.J., Phansopa C., Honma K., Urbanowicz P.A., Spencer D.I.R., Pratten J., Bradshaw D., Sharma A., Stafford G. (2019). Characterization of *Porphyromonas gingivalis* sialidase and disruption of its role in host-pathogen interactions. Microbiology.

[36] Franca R.D.G., Vieira A., Carvalho G., Oehmen A., Pinheiro H.M., Crespo M.T.B., Lourenco N.D. (2020). *Oerskovia paurometabola* can efficiently decolorize azo dye Acid Red 14 and remove its recalcitrant metabolite. Ecotoxicol. Environ. Saf..

[37] Ghazaei C., Ahmadi M., Jazani N.H. (2010). Detection of neuraminidase activity in *Pseudomonas aeruginosa* PAO1. IJBMS.

[38] Gualdi L., Hayre J.K., Gerlini A., Bidossi A., Colomba L., Trappetti C., Pozzi G., Docquier J.-D., Andrew P., Ricci S. (2012). Regulation of neuraminidase expression in *Streptococcus pneumoniae*. BMC Microbiol..

[39] Engibarov S., Eneva R., Abrashev I. (2015). Neuraminidase (sialidase) from *Aeromonas* sp. strain A40/02—Isolation and partial purification. Ann. Microbiol..

[40] Therit B., Cheung J.K., Rood J.I., Melville S. (2015). NanR, a transcriptional regulator that binds to the promoters of genes involved in sialic acid metabolism in the anaerobic pathogen *Clostridium perfringens*. PLoS ONE.

[41] Abrashev I., Orozova P. (2006). *Erysipelothrix rhusiopathiae* neuraminidase and its role in pathogenecity. Z. Nat..

[42] Li J., McClane B. (2014). The sialidases of *Clostridium perfringens* type D strain CN3718 differ in their properties and sensitivities to inhibitors. Appl. Environ. Microbiol..

[43] Jost B.H., Songer G.J., Billington S.J. (2001). Cloning, expression and characterization of a neuraminidase gene from *Arcanobacterium pyogenes*. Infect. Immun..

[44] Schwerdtfeger S.M., Melzig M.F. (2010). Sialidases in biological systems. Pharmazie.

[45] Copley R.R., Russell R.B., Ponting C.P. (2001). Sialidase-like Asp-boxes: Sequence-similar structures within different protein folds. Protein Sci..

[46] Crennel S., Garman E., Laver G., Vimr E., Taylor G. (1994). Crystal structure of *Vibrio cholerae* neuraminidase reveals dual lectin-like domains in addition to the catalytic domain. Structure.

[47] Vimr E.R., Kalivoda K.A., Deszo E.L., Steenbergen S.M. (2004). Diversity of microbial sialic acid metabolism. Microbiol. Mol. Biol. Rev..

[48] Nicholas K.B. (1997). Genedoc: A tool for editing and annotating multiple sequence alignments. Embnew. News..

[49] Waterhouse A., Bertoni M., Bienert S., Studer G., Tauriello G., Gumienny R., Heer F.T., de Beer T.A.P., Rempfer C., Bordoli L. (2018). SWISS-MODEL: Homology modelling of protein structures and complexes. Nucleic Acids Res..

[50] Uchida Y., Tsukada Y., Sugimori T. (1977). Distribution of neuraminidase in *Arthrobacter* and its purification by affinity chromatography. J. Biochem..

[51] Abrashev I., Velcheva P., Nikolov P., Kourteva J. (1980). Substrate for Colorimetric Determination of Enzyme Activity. Bulgaria Patent.

[52] Bradford M.M. (1976). A rapid and sensitive method for the quantitation of microgram quantities of protein utilizing the principle of protein-dye binding. Anal. Biochem..

[53] Laemmli U.K. (1970). Cleavage of structural proteins during the assembly of the head of bacteriophage T4. Nature.

[54] Chevallet M., Luche S., Rabilloud T. (2006). Silver staining of proteins in polyacrylamide gels. Nat. Protoc..

[55] Lineweaver H., Burk D. (1934). Determination of enzyme dissociation constants. J. Am. Chem. Soc..

